# Dominant-negative p53-overexpression in skeletal muscle induces cell death and fiber atrophy in rats

**DOI:** 10.1038/s41419-022-05160-6

**Published:** 2022-08-17

**Authors:** Henning T. Langer, Agata A. Mossakowski, Rasheed Sule, Aldrin Gomes, Keith Baar

**Affiliations:** 1 Department of Physiology and Membrane Biology, University of California, Davis, CA USA; 2 Division of Endocrinology, Weill Department of Medicine, Weill Cornell Medicine, New York, NY USA; 3 Neurobiology, Physiology and Behavior, University of California, Davis, CA USA; 4 Charité - Universitätsmedizin Berlin, corporate member of Freie Universität Berlin, Humboldt-Universität zu Berlin, and Berlin Institute of Health, Berlin, Germany; 5 VA Northern California Health Care System, Mather, CA USA

**Keywords:** Proteasome, Phosphorylation, Contractile proteins

## Abstract

The tumor suppressor p53 is thought to play a key role in the maintenance of cell size and homeostasis, but relatively little is known about its role in skeletal muscle. Based on its ability to suppress cell growth, we hypothesized that inhibiting the function of wild-type p53 through the overexpression of a dominant-negative p53 mutant (DDp53) could result in muscle fiber hypertrophy. To test this hypothesis, we electroporated adult rat tibialis anterior muscles with DDp53 and collected the tissue three weeks later. We confirmed successful overexpression of DDp53 on a histological and biochemical level and found pronounced changes to muscle architecture, metabolism, and molecular signaling. Muscle mass, fiber cross-sectional area, and fiber diameter significantly decreased with DDp53 overexpression. We found histopathological changes in DDp53 transfected muscle which were accompanied by increased levels of proteins that are associated with membrane damage and repair. In addition, DDp53 decreased oxidative phosphorylation complex I and V protein levels, and despite its negative effects on muscle mass and fiber size, caused an increase in muscle protein synthesis as assessed via the SUnSET technique. Interestingly, the increase in muscle protein synthesis was concomitant with a decrease in phospho-S6K1 (Thr389). Furthermore, the muscle wasting in the DDp53 electroporated leg was accompanied by a decrease in global protein ubiquitination and an increase in proteasome activity. In conclusion, overexpression of a dominant-negative p53 mutant in skeletal muscle results in decreased muscle mass, myofiber size, histological muscle damage, a metabolic phenotype, and perturbed homeostasis between muscle protein synthesis and degradation.

## Introduction

In humans, skeletal muscle accounts for approximately 40% of body weight as well as 70% of body protein [1]. Together with tendons and bones it enables locomotion, but importantly also acts as reservoir for glucose, fat, and amino acids. The loss of skeletal muscle mass and strength is independently associated with a decrease in quality of life, life span, and health span [2–4]. Unfortunately, there is currently no available drug treatment for muscle wasting. With the advent of molecular biology, numerous potential therapeutic targets regulating muscle size have emerged. Prominent examples of negative regulators of muscle mass include E3 ubiquitin-ligases or signaling through TGFβ family members such as BMP or myostatin [5, 6]. We have previously identified a class of proteins that we called molecular brakes, which slow the rate of muscle hypertrophy in response to extreme growth stimuli [7]. Many regulators of cell size are not unique to muscle cells but are conserved across different tissues. In other tissues, growth, especially uncontrolled growth, is pathological and the resulting cancers are one of the main scientific and clinical challenges of our time. However, many of the cell growth and differentiation pathways discovered in cancer cells could promote improved mass and function in muscle.

The protein p53 was the first tumor suppressor protein characterized and is altered in most cancers [8]. p53 gets activated in response to stresses such as genotoxicity, oncogenes, hypoxia, starvation, or aging and drives cell cycle arrest and apoptosis [9]. For the containment of tumors, these are desirable traits and explain why certain species with a greater number of TP53 copies in the genome are particularly resilient against cancer, and why mutations in TP53 that impair its function are associated with a greater occurrence of cancer [9, 10]. However, outside of tumors and especially in post-mitotic tissues that carry little to no potential for the development of cancer, the role of p53 is less defined. In skeletal muscle, most investigations into p53 have surrounded its effect on mitochondrial function, where analyses of whole body p53 knockout mice have revealed that p53 is important for mitochondrial biogenesis, mitochondrial DNA content, the adaptive response to an acute bout of exercise as well as aerobic exercise capacity [11–13]. The loss of p53 specifically in skeletal muscle, however, did not result in differences in mitochondrial and metabolic gene expression or protein levels [14]. Similarly, the absence of p53 in skeletal muscle had neither beneficial nor deleterious effects during aging [15]. Therefore, the role of p53 in the regulation of skeletal muscle mitochondrial mass and function is equivocal.

The result of mutation of p53 can be classified as: (1) loss of function, (2) a trans-dominant effect of mutant over wild-type p53, and/or (3) a gain of oncogenic potential [16]. Induction of a dominant-negative p53 (DDp53) in heterozygous p53 deficient mice has been shown to decrease p53 activity and increase tumor incidence in wild-type mice but not p53 null mice, supporting the idea that DDp53 is more likely to have an effect in the presence of wild type p53 [17]. In line with this, in human families with the Li-Fraumeni Syndrome, individuals showed an earlier age of diagnosis when having a missense mutation compared to those with a mutation that results in truncation of the protein [18]. Such dominant-negative effects of DDp53 on wild-type p53 are thought to be a result of heterotetramerization [19], the binding of DDp53 to transcriptional cofactors required for wild-type p53 function [20] or both, eventually leading to an inability of wild type p53 to bind to DNA and exert its growth suppressing and apoptosis-inducing effects [21].

While the effects of DDp53 in tumors and tumor hosting tissues are thoroughly investigated, there is a sparsity of information on more tumor-resilient tissues such as skeletal muscle. Therefore, we aimed to investigate the effect of DDp53 on skeletal muscle homeostasis. As there is an unanswered need for therapeutical interventions that allow muscle to grow and reverse wasting, understanding the role of a highly preserved growth suppressor like p53 could allow for important insights. We hypothesized that local overexpression of DDp53 would decrease muscle protein degradation and increase muscle size.

## Methods

### Animals

All procedures were approved by the Institutional Animal Care and Use Committee (IACUC) of the University of California, Davis, which is an AAALAC-accredited institution. Animal housing was in accordance with recommendations of the Guide for the Care and Use of Laboratory Animals. Animals were housed in 12:12 h light-dark cycles, fed ad libitum in a conventional vivarium, and were specific pathogen-free. Female Fischer 344-Brown Norway rats at the age of 10 months were acquired from the National Institute of Aging rodent core to study the effect of DDp53 on skeletal muscle (*n* = 12). The sample size was chosen based on pilot experiments and effect size estimates.

### Electroporation protocol

Rats were anesthetized using inhaled isoflurane (3%) and the electroporation of DNA plasmids was performed as previously described [22, 23]. Briefly, after a 2 h pre-treatment with 0.4 units/μl of hyaluronidase, 20 μg of plasmid DNA was injected into the tibialis anterior (TA) muscle, the hindlimbs were placed between two paddle electrodes and subjected to 8 pulses (20 ms) of 160 V/cm using an ECM-830 electroporator (BTX Harvard Apparatus, Cambridge, MA, USA). All animals (*n* = 12) were injected with both a dominant-negative p53 plasmid (T7-p53DD-pcDNA3) that was a gift from William Kaelin (Addgene plasmid # 25989) [24] into the ipsilateral TA, and a mCherry control plasmid into the contralateral control TA in a random order. Pre-treatment with hyaluronidase allows for increased transfection levels in skeletal muscle without further muscle damage during the electroporation process (McMahon et al. 2001). Twenty-one days after transfection the animals were collected according to the protocol described below.

### Muscle collection

Animals were anesthetized using inhaled isoflurane (3%) and the tibialis anterior muscles of both sides were collected, pinned down on cork at resting length, submerged in liquid nitrogen-cooled isopentane until fully frozen, and then transferred to dry ice. Equal-sized sections of the mid-belly of the tibialis anterior muscle were later cut out in the cooling chamber of a cryostat (Leica CM 3050S, Leica Microsystems, IL USA) and placed on optimal cutting temperature compound (VWR, CA, USA) for muscle histology processing as described below.

### Histology

Following blocking, serial cross-sections were cut at 10 μm. For hematoxylin and eosin (H&E) staining, sections were consecutively submerged in 90% ethanol and then tap water. 400 µL Mayer’s hemalum solution (Merck, Darmstadt, Germany) was applied per slide and left to incubate for 6 min. Slides were then blued under running lukewarm tap water before being dipped in tap water for another 8 min. 300 µL 0.5% aqueous eosin γ-solution (Merck, Darmstadt, Germany) were applied to each slide for 1 min. Sections were dehydrated in sequential steps using 70%, 90%, and 100% ethanol. Sections were submerged in Xylene, air dried, and mounted with DPX Mountant for histology (Sigma, Darmstadt, Germany). For immunohistochemistry, TA sections were fixed in acetone, washed, and blocked with 5% natural goat serum (NGS) for 30 min. For determination of fiber size, we incubated slides with a polyclonal laminin antibody (rabbit, IgG (H + L), 1:500 in NGS) over night at 4 °C. On day two, samples were incubated in the secondary antibody (goat-anti-rabbit Alexa Fluor 647) (Cell Signaling Technology, Danvers, MA) (catalog #4414) for 30 min at room temperature. The anti-laminin antibody was purchased from Sigma Aldrich (St Louis, Missouri) (catalog #L9393).

Slides were imaged using a Leica DMi8 inverted microscope using the HC PL FLUOTAR 10x/0.32 PH1 objective (Leica Microsystems, Wetzlar, Germany). For comparative analysis, exposure length remained fixed for all samples. For all stains, overlapping images were stitched together such that the entire muscle could be analyzed. Muscle fiber properties were analyzed using FIJI and SMASH (MATLAB) as described previously [25, 26]. For the assessment of Feret’s diameter, the shortest possible distance between two sides of the myofiber was determined to account for the possibility of an uneven plane of the cryosection that may have confounded analysis of the fiber cross-sectional area. The investigators were blinded for the histological analysis.

### Immunoblotting

Frozen powdered tibialis anterior muscles were homogenized in 200 µL sucrose lysis buffer (SLB; 50 mM Tris pH 7.5, 250 mM sucrose, 1 mM EDTA, 1 mM EGTA, 1% Triton X-100, 1% protease inhibitor complex) on a vortexer for 60 min at 4 °C. Following centrifugation at 10,000 *g* for 10 min, the supernatant was collected. Protein concentrations were determined in triplicate using the DC protein assay (Bio-Rad, Hercules, CA, USA). Sample concentrations were adjusted using SLB. Following dilution in Laemmli sample buffer (LSB), samples were denatured at 100 °C for 5 min. Protein (10–30 µg per lane) was loaded on 4–15% Criterion TGX Stain-free gels (Bio-Rad), run for 45 min at 200 V, and visualized after a UV-induced 1-minute reaction to produce fluorescence. Following quantification, proteins were transferred to nitrocellulose or polyvinylidene difluoride (PVDF) membrane at 100 V for 30-60 min, depending on the size of the protein of interest. Efficient transfer was confirmed using Ponceau S staining of the membrane. Membranes were then air dried and directly incubated with the primary antibody, or washed and blocked in 1% fish skin gelatin dissolved in Tris-buffered saline with 0.1% Tween-20 (TBST) for 1 h, rinsed, and then incubated with the primary antibody (1:1000 in TBST) overnight at 4 °C. The next day, membranes were washed and incubated with HRP-conjugated secondary antibodies at 1:5000 (goat) (catalog #31402, Pierce, Rockford, IL) to 1:10 000 (mouse, rabbit) (catalog #7076 and 7074, Cell Signaling Technology, Danvers, MA) in 1% skim milk-TBST for 1 h at room temperature. Immobilon Western Chemiluminescent HRP substrate (Millipore, Hayward, CA, USA) was then applied to the membranes for protein visualization by chemiluminescence. Image acquisition and band quantification was performed using the ChemiDoc MP System and Image Lab 5.0 software (Bio-Rad). Protein levels of each sample were calculated as band intensities relative to total protein as described previously [27]. All samples of both groups (i.e., contralateral control and ipsilateral DDp53 TA) were run on the same gel for each protein probed; images of the bands in the figures are representative, with biological replicates shown for each treatment.

The following antibodies were used in this study at a concentration of 1 to 1000. Cell Signaling (Cell Signaling Technology, Danvers, MA): T7-tag (#13246, lot 1), phospho-p70-S6 Kinase 1 (S6K1) (Thr389) (#9205; lot 16), phospho-ribosomal protein S6 (rS6) (Ser240/244) (#5364), phospho-eukaryotic elongation factor 2 (eEF2) (Thr56) (#2331), forkhead box O3 (FOXO3a) (#2497, lot 8), microtubule-associated proteins 1A/1B light chain 3B (LC3B) (#2775; lot 10), autophagy related 7 (Atg 7) (#8558), heat shock protein β-1 (HSP27) (#2402; lot 8), phospho-Unc-51 like autophagy activating kinase 1 (ULK1) (Ser757) (#14202; lot 1), sequestosome 1 (p62) (#5114; lot 4), nuclear factor κ B (NF-kB) (Ser536) (#3033; lot14), annexin A2 (#8235; lot 2), caspase 3 (#9662), ubiquitin (#3933, lot5), glyceraldehyde 3-phosphate dehydrogenase (GAPDH) (#5174); Santa Cruz (Santa Cruz Biotechnology Inc, Dallas, TX): dystrophin (#365954; lot E2711), dysferlin (#16635; lot H162), glucose transporter type 4 (GLUT4) (#53566; lot 2415), muscle LIM protein/cysteine and glycine-rich protein 3 (mLIM) (#166930; lot E2814); Millipore Sigma (Merck Group): insulin receptor substrate 1 (IRS1) (#06-248; lot 2465193), puromycin (MABE343); Developmental Studies Hybridoma Bank (University of Iowa, Iowa City, IA): myosin heavy chain—Slow (#BA-D5), procollagen type 1a1 (Col1a1) (SP1-D8); Abcam (Abcam Inc, Eugene, OR): total oxidative phosphorylation (OXPHOS) (MS604-300).

### Muscle protein synthesis

Global muscle protein synthesis was assessed using the SUrface SEnsing of Translation (SUnSET) method as described previously [28]. Briefly, puromycin is a structural analogue of the tyrosine aminoacyl-transfer RNA and gets incorporated into elongating peptide chains via the formation of a peptide bond [29]. Incorporation of puromycin, therefore, results in the premature termination and release of the peptide chain from the ribosome. The injection of puromycin and subsequent analysis via immunoblot has been shown to be a valid method to assess muscle protein synthesis comparable to stable isotope tracing [30]. Puromycin was dissolved in sterile saline (0.9% NaCl) and delivered via i.p. injection (0.02 μmol puromycin × g^–1^ body weight) 30 min prior to muscle collection. Puromycin-truncated peptides, reflecting the rate of global muscle protein synthesis, were analyzed by western blot as described above.

### Lysate 26S proteasome activity assay

The 26 S proteasome assay was carried out as described previously [31–33]. A total volume of 100 μl was incubated in 96 well plates containing 100 μM ATP in 26S buffer and 20 μg of protein supernatants. Assays were initiated by addition of fluorescently labeled substrate: succinyl-Leu-Leu-Val-Tyr-7-amido-4-methylcoumarin (Suc-LLVY-AMC), Boc-Leu-Ser-Thr-Arg-AMC (Boc-LSTR-AMC, Bachem), and Z-Leu-Leu-Glu-AMC (Z-LLE-AMC) for chymotrypsin- (β5), trypsin- (β2) and caspase- (β1) like activity measurements, respectively. The final concentration of substrate in each assay was 100 μM.

These substrates are cleaved by the proteasome, releasing free 7-amino-4-methylcoumarin (AMC) which was then measured spectrofluorometrically using an Infinite M1000 PRO fluorometer (Tecan, Männedorf, Switzerland) at an excitation wavelength of 390 nm and an emission wavelength of 460 nm. Fluorescence was measured at 15-min intervals for 2 h. Each assay was conducted in the absence and presence of the specific proteasomal inhibitor bortezomib (LC labs, Woburn, MA), 10 μM for β5 chymotrypsin-like activity, and 100 μM for β2 trypsin-like activity, and β1 caspase-like activity. All assays were assessed for linearity, and the results of either of the two time-points with the highest proteasome activity in the linear range (i.e., 75 min and 90 min) were used for analysis.

### Statistics

Data analysis was carried out using Graphpad Prism 9.2.0 (GraphPad Software, San Diego, CA). A paired *t*-test was used to assess group differences for muscle mass, fiber type, and protein levels. Correlations between T7-tag protein levels and other variables were carried out through a simple linear regression analysis exclusively in the DDp53 electroporated leg. Goodness of fit was calculated as R squared. The alpha level was set at *p* = 0.05; *p* values < 0.05 were deemed statistically significant, and *p* values between 0.05 and 0.1 were considered trends.

## Results

### Experimental design and DDp53 overexpression in tibialis anterior muscle

**Fig. 1:**
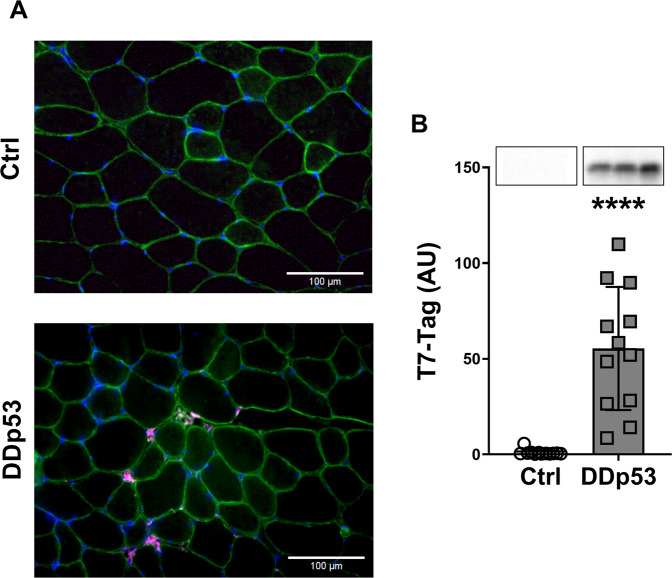
Histological localization and protein levels of DDp53. **A** Immunohistological representation of DDp53 in the contralateral control leg (left) and the leg electroporated with T7-tagged DDp53. DDp53 was detected via T7-tag (purple) and was mainly located in the interstitium of the ipsilateral tibialis anterior, while it was absent in the contralateral control leg. Sarcolemma shown via anti-laminin-staining (green). Nuclear staining via DAPI (blue). **B** Western blot confirmed the successful overexpression of DDp53 via increased T7-Tag protein levels in the ipsilateral compared to the contralateral control leg. Individual data points represent individual animals. Squares represent the leg that was electroporated with DDp53 and circles the contralateral control leg of the same animals. Western blot images show three representative bands from each condition. Both representative pictures stem from the same membrane and exposure time. All western blots were normalized to total protein per lane. Original, unaltered images of all membranes and the gels that they were normalized to can be found in the data supplement. *n* = 12, **** indicates a *p*-value of <0.0001.

To explore the effect of dominant-negative p53 (DDp53) on skeletal muscle, we electroporated the DDp53 plasmid into the ipsilateral tibialis anterior of twelve rats, while electroporating mCherry into the contralateral control leg of the same animals. The animals were collected three weeks after electroporation and the tissue was weighed and frozen for histological and biochemical analysis. The DDp53 plasmid was tagged with a T7-epitope tag, which allowed us to identify its localization both histologically and biochemically. We found DDp53 to be predominantly present in the interstitial space between fibers in the ipsilateral tibialis anterior but not in the contralateral control muscle (Fig. 1A). The localization outside of myofibers is consistent with previous reports on p53 in skeletal muscle [14], possibly displaying residual protein after successful fiber degradation. To further confirm overexpression of DDp53 in the ipsilateral tibialis anterior we performed western blotting and found that T7-tag protein levels were 53-fold as high as in the contralateral control leg (*p* < 0.0001) (Fig. 1B).

### Local DDp53 overexpression results in decreased muscle mass, fiber size and fiber damage

**Fig. 2:**
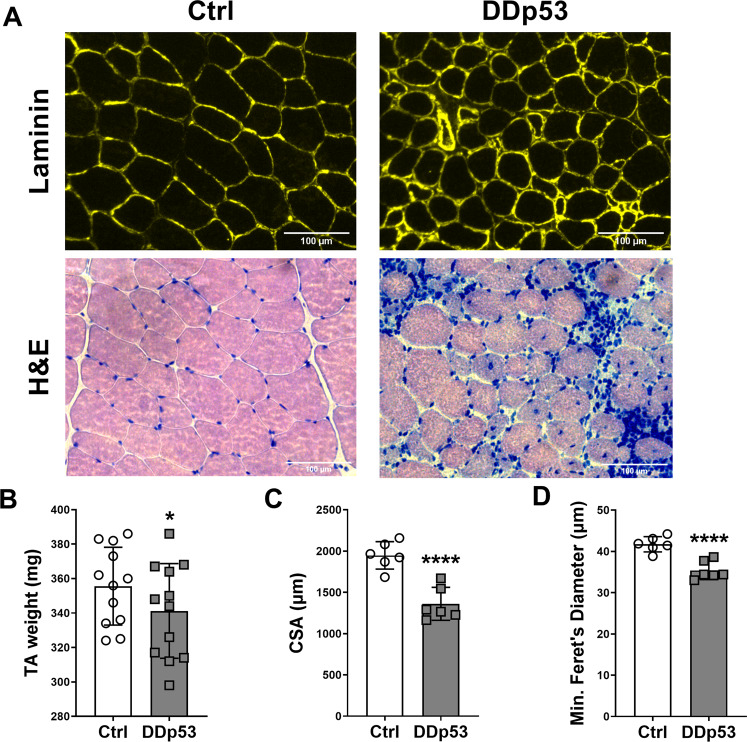
Changes to muscle mass, fiber size and health after DDp53 electroporation. **A** Immunohistological assessment of myofiber cross-sectional area via laminin staining (Laminin, upper row), and hematoxylin and eosin staining (H&E, lower row) to assess fiber integrity indicated central nuclei, regenerating fibers, macrophage infiltration, and fiber death. **B** Muscle mass in the DDp53 tibialis anterior was significantly reduced three weeks after electroporation. **C** Fiber cross sectional area was significantly reduced in DDp53 three weeks after electroporation. **D** Fiber diameter was significantly reduced in DDp53 three weeks after electroporation. *n* = 6 to 12, * indicates a *p*-value of <0.05, **** indicates a *p*-value of <0.0001.

To investigate morphological changes to skeletal muscle with DDp53 overexpression, we examined the tibialis anterior macroscopically by assessing muscle mass and microscopically via histological analysis. Electroporation of the DDp53 plasmid into tibialis anterior muscle caused pronounced changes on the histopathological level: HE-staining revealed increased central nuclei, regenerating fibers, necrotic fibers, macrophage infiltration, and fibrosis with DDp53 compared to the control leg (Fig. 2A). These alterations led to a 14 mg lower muscle mass in the DDp53 tibialis anterior compared to the mCherry electroporated contralateral control tibialis anterior of the same animals (341 ± 28 versus 356 ± 23 mg, respectively) (*p* < 0.05) (Fig. 2B). To improve our understanding of the effect of chronic DDp53 overexpression on individual muscle fiber size, we determined muscle fiber cross-sectional area and fiber diameter. Mean muscle fiber cross-sectional area in the DDp53 tibialis anterior decreased more than 30% compared to the contralateral control muscle (1360 ± 200 to 1947 ± 166 µm, respectively) (*p* < 0.0001) (Fig. 2C). Similarly, minimal Feret’s diameter decreased from 42 ± 2 in the control leg to 35 ± 2 µm in the DDp53 treated leg (*p* < 0.0001) (Fig. 2D).

### Changes to proteins responsible for modulating the muscle cytoskeleton

**Fig. 3:**
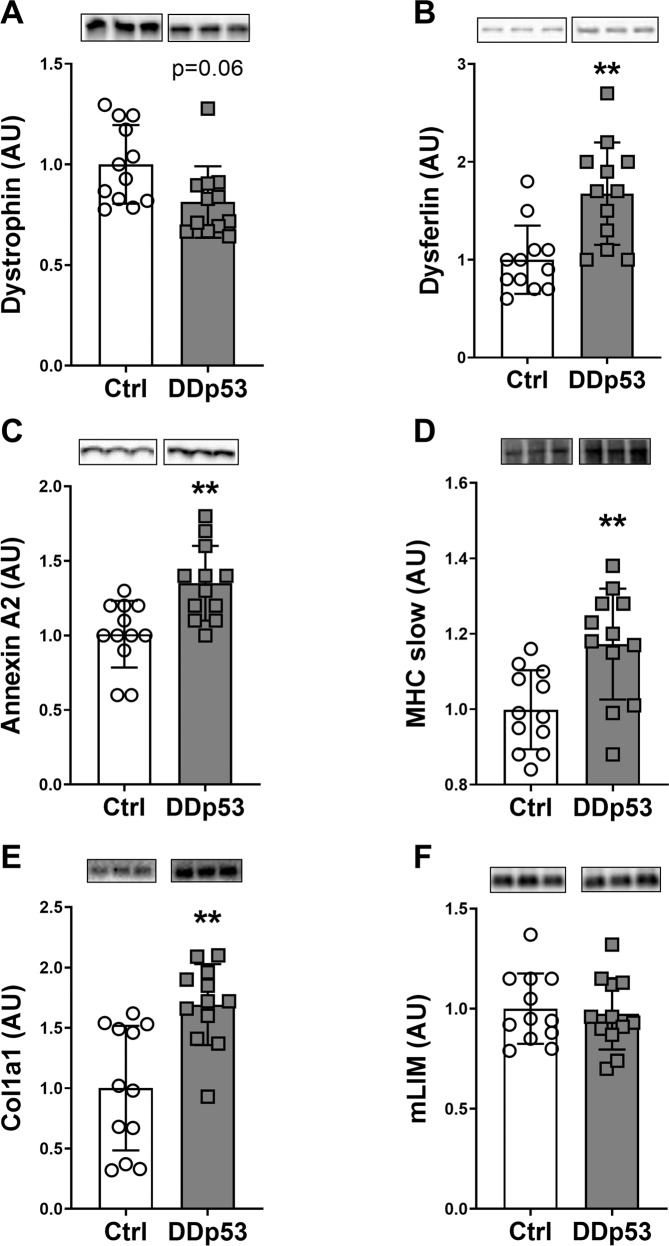
Proteins associated with cytoskeletal integrity and fibrosis are affected by DDp53 overexpression. **A** Dystrophin levels tended to be reduced three weeks after DDp53 electroporation. B The membrane-damage activated protein dysferlin was increased with DDp53 electroporation. **C** Similarly, annexin A2 levels increased with DDp53. **D** Myosin heavy chain type I (slow isoform/MHCs) protein levels was elevated in the DDp53 electroporated muscle compared to the control leg. **E** Pro-collagen (proCol1α1) synthesis was increased in the DDp53 leg compared to the control leg. **F** Muscle LIM (mLIM) protein levels were unchanged with DDp53. Individual data points represent each animal. Squares represent the leg that was electroporated with DDp53, circles the contralateral control leg of the same animals. Western blot images show three representative bands from each condition. Both representative pictures stem from the same membrane and exposure time. All western blots were normalized to total protein per lane. Original, unaltered images of all membranes and the gels that they were normalized to can be found in the data supplement. *n* = 12, * indicates a *p*-value of <0.05 and ** indicates a *p*-value of <0.01.

Because there was a decrease in fiber size and a histological phenotype consistent with muscle damage, we next investigated how DDp53 affected structural proteins that are necessary for force transfer and muscle repair. Dystrophin is one of the largest proteins of the human body and essential for muscle membrane integrity and function [34]. Lack of dystrophin causes muscular dystrophy and atrophy, with increased central nuclei, regenerating fibers, necrotic fibers, macrophage infiltration, and fibrosis [35]. Overexpression of DDp53 resulted in a 19% decrease in dystrophin protein levels that trended towards significance (*p* = 0.06) (Fig. 3A). Since even small changes in dystrophin could result in increased susceptibility of the muscle membrane to injury, we measured proteins associated with sarcolemma repair. One of such molecules is dysferlin which together with annexin A2 is essential for membrane repair [36, 37]. We previously reported that muscle-damaging exercise and a concomitant increase in membrane lesions lead to an increased expression of dysferlin [38]. In the study at hand, membrane injury-associated dysferlin protein levels increased significantly with DDp53, reaching 68% higher levels than in the control leg (*p* < 0.01) (Fig. 3B). Similarly, the sarcolemma repair protein annexin A2 increased 34% with DDp53 compared to the control leg (*p* < 0.01) (Fig. 3C). Together, the decrease in dystrophin and the increase in dysferlin and annexin A2 suggest that DDp53 overexpression decreases sarcolemma stability, resulting in increased susceptibility to injury and upregulation of membrane repair proteins such as dysferlin and annexin A2. Alternatively, the decrease in dystrophin could also be the result of a specific loss of sarcolemmal proteins as part of the muscle fiber atrophy observed with DDp53 overexpression. To investigate whether contractile protein content was affected by DDp53, we looked at myosin heavy chain. Levels of the slow myosin heavy chain (MHCs) increased by 18% in the DDp53 treated TA compared to the control TA (*p* < 0.01) (Fig. 3D). Rather than a transformation from fast- to slow twitch heavy chain isoforms, this is likely a function of the fast type 2 fibers being damaged disproportionately [39]. Such a decrease in fast myosin heavy chain would lead to a relative increase in MHCs in the total protein pool. To determine whether the increase in damage and repair of DDp53 overexpressing muscle would lead to increased connective tissue infiltration as is seen in muscular dystrophies, we measured protein levels of the soluble collagen fraction pro-collagen -α1(I) (Col1α1). Consistent with the increase in connective tissue that we identified histologically (Fig. 2A), proCol1α1 protein increased 69% (*p* < 0.01) in the DDp53 TA compared to the control TA (Fig. 3E). Finally, CSRP3 or muscle-specific LIM (mLIM) has been reported to have essential roles in myogenesis and differentiation [40], as well as to interact with cytoskeletal proteins such as actin [41] and sense mechanical stress at the z-disc [42]. We have recently found mLIM to be robustly increased in another model of muscular dystrophy (‘desminopathy’) and in response to muscle-damaging exercise [43]. In the study at hand, levels of mLIM did not differ significantly between the DDp53 and the contralateral control leg (*p* = 0.75) (Fig. 3F). Interestingly, however, we found a modest but significant correlation between DDp53 and mLIM protein levels when looking exclusively at the DDp53 electroporated leg (Supplemental Fig. 1). This would suggest that while the relative content of mLIM in the total protein pool of DDp53 overexpressing muscle is unchanged compared to contralateral control muscle, DDp53 still has a modest effect on mLIM that could be a consequence of the deleterious impact of DDp53 on sarcolemma integrity.

### The impact of DDp53 on glucose transport and oxidative phosphorylation

**Fig. 4:**
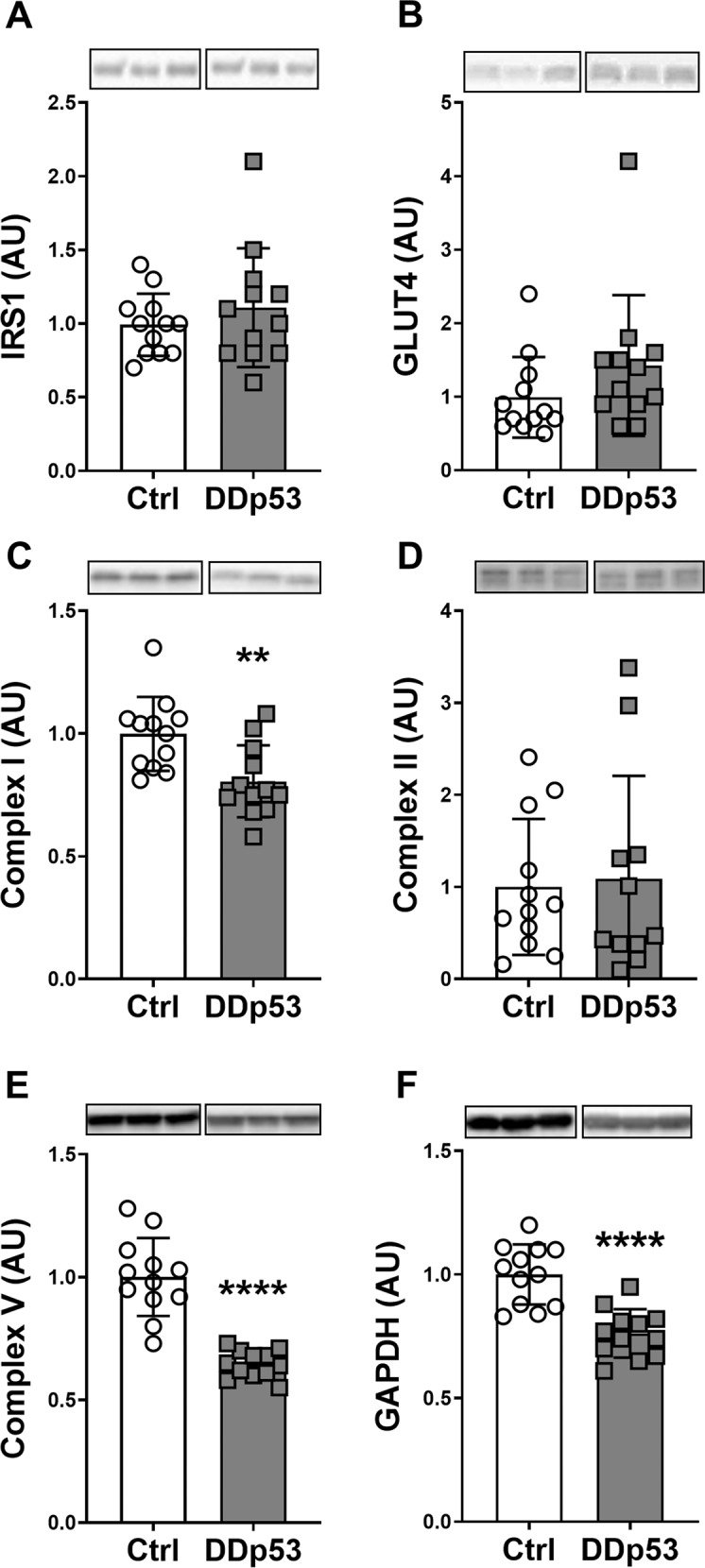
Changes to oxidative phosphorylation and glucose metabolism with DDp53 in skeletal muscle. **A** Total IRS1 protein levels did not change with DDp53 electroporation. **B** Glucose transporter GLUT4 protein levels did not change with DDp53 electroporation. **C** Mitochondrial complex I (NDUFB8) protein levels were significantly decreased with DDp53 in tibialis anterior muscle. **D** Mitochondrial complex II (SDHB) protein levels were unchanged with DDp53. **E** Mitochondrial complex V (ATP5A) protein levels were significantly reduced after DDp53 electroporation compared to control muscle. **F** GAPDH protein levels were significantly reduced with DDp53 compared to the control leg. Individual data points represent the results from a single animal. Squares represent the leg that was electroporated with DDp53, circles the contralateral control leg of the same animals. Western blot images show three representative bands from each condition. Both representative pictures stem from the same membrane and exposure time. All western blots were normalized to total protein per lane. Original, unaltered images of all membranes and the gels that they were normalized to can be found in the data supplement. *n* = 12, ** indicates a *p*-value of <0.01 and **** indicates a *p*-value of <0.0001.

Previous research into the role of p53 in skeletal muscle has suggested a pivotal role in mitochondrial function. Whole-body knockouts of p53 found that mitochondrial DNA content, enzyme activity, and biogenesis were negatively affected in skeletal muscle, resulting in decreased aerobic capacity and running performance [11–13]. Therefore, we next explored the effect of DDp53 on key regulators of muscle metabolism. Insulin receptors substrates (IRS) are essential conductors of the insulin pathway [44], and decreased protein levels seem associated with decreased anabolic signaling and insulin sensitivity [45, 46]. Total IRS1 protein was not different between the TA with DDp53 and the mCherry control TA (*p* = 0.39) (Fig. 4A). The primary transporter of insulin-dependent and independent glucose uptake into skeletal muscle is GLUT4 [47–49]. GLUT4 protein also did not change (*p* = 0.26) in response to the DDp53 treatment compared to the control (Fig. 4B). To investigate whether local overexpression of DDp53 would produce similar effects on mitochondrial function as the whole-body knockout, we looked at several proteins of the electron transport chain. DDp53 electroporation significantly decreased complex I (19%; *p* < 0.01) and V (36%; *p* < 0.0001) compared to the contralateral control leg, indicating that impairing wild-type p53 function through DDp53 had inhibitory effects on mitochondrial function (Fig. 4C, E). Complex II protein levels, however, were not different (*p* = 0.81) between the groups (Fig. 4D). Finally, we looked at glyceraldehyde 3-phosphate dehydrogenase (GAPDH) which is commonly used as a housekeeping protein but is also an important glycolytic enzyme. Here, we detected that GAPDH levels in the DDp53 leg were 24% lower (*p* < 0.0001) than the control leg (Fig. 4F). Together, these data suggest that DDp53 overexpression has a modest effect on skeletal muscle substrate metabolism that predominantly concerns mitochondrial enzymes.

### Muscle protein synthesis, translation initiation, and anabolic signaling

**Fig. 5:**
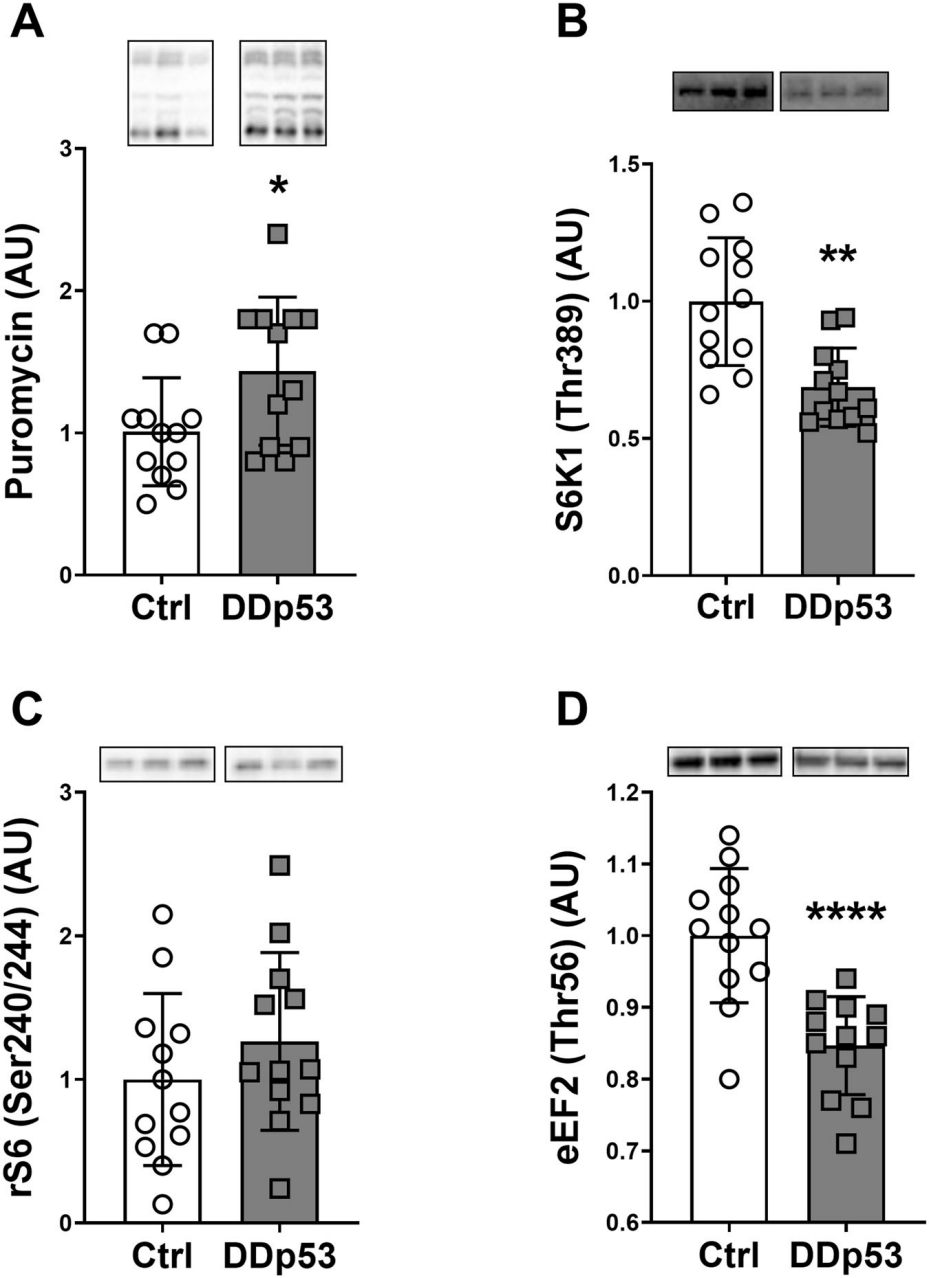
mTORC1 signaling and muscle protein synthesis. **A** Puromycin levels were increased in the DDp53 electroporated tibialis anterior muscle compared to the contralateral control leg. **B** Simultaneously, phospho-S6K1 (Thr389) levels were significantly decreased in the DDp53 leg. **C** At the same time, downstream target phospho-rS6 (Ser240/244) was unchanged between the legs. **D** Phospho-eEF2 (Thr56) was significantly decreased after DDp53 electroporation compared to the control. Individual data points represent the results from each animal. Squares represent the leg that was electroporated with DDp53, circles the contralateral control leg of the same animals. Western blot images show three representative bands from each condition. Both representative pictures stem from the same membrane and exposure time. All western blots were normalized to total protein per lane. Original, unaltered images of all membranes and the gels that they were normalized to can be found in the data supplement. *n* = 12, * indicates a *p*-value of <0.05, ** indicates a *p*-value of <0.01 and **** indicates a *p*-value of <0.0001.

To obtain insights into the physiological changes that drove the decrease in muscle mass and fiber size with DDp53 overexpression, we measured muscle protein synthesis by injecting rats with puromycin prior to tissue collection. Puromycin is an aminoacyl transfer RNA analogue that is incorporated into nascent peptide chains terminating their elongation [29]. The accumulation of puromycin-conjugated peptides can then be detected via antibody using the surface sensing of translation (SUnSET) method and this reflects global protein synthesis rates in cells and various tissues, including skeletal muscle [30, 50, 51]. DDp53 overexpression caused a 43% increase (*p* < 0.05) in muscle protein synthesis as assessed via puromycin incorporation (Fig. 5A). To learn more about the molecular signals underlying this observation, we looked at different proteins within the canonical mTORC1 pathway known to regulate tissue anabolism. Despite the increase in muscle protein synthesis, the mTORC1 target S6K1 (Thr389), was 31% (*p* < 0.01) less phosphorylated in the DDp53 TA compared to the contralateral control TA (Fig. 5B). Phosphorylation of ribosomal protein S6 (Ser240/244), a substrate of S6K1, did not change (*p* = 0.34) with DDp53 overexpression (Fig. 5C). In contrast to the decrease in S6K1 phosphorylation, which should increase eEF2 kinase activity and eEF2 phosphorylation, we found a 15% decrease (*p* < 0.0001) in phosphorylated eEF2 (Thr56) in the DDp53 treated TA compared to the contralateral TA (Fig. 5D). These contrasting data will be more thoroughly delineated in the discussion section below.

### Degradation, inflammation, and cell death

**Fig. 6:**
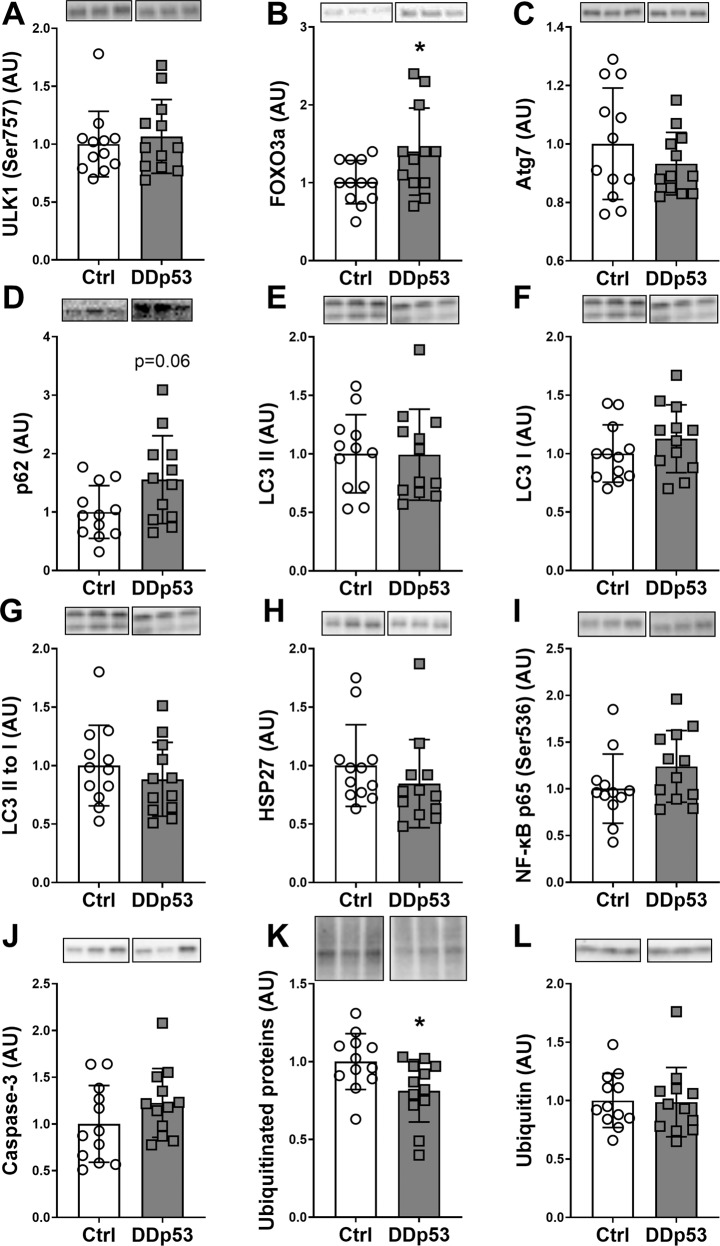
Degradation, inflammation, and cell death after DDp53 overexpression. **A** Phospho-ULK1 (Ser757) was unchanged following DDp53 electroporation. **B** Total FOXO3a protein levels were increased in the electroporated DDp53 tibialis anterior muscle compared to the control. **C** Atg7 was unchanged between both sides. **D** Autophagy substrate p62 trended to be increased in the DDp53 leg compared to the control side. **E**–**G** LC3 I, II and the LC3 II to I ratio was not significantly different at three weeks of DDp53 overexpression. **H** HSP27 was not different between the legs. **I** Phospho-p65 (Ser536) was not different between the DDp53 and the control leg. **J** Caspase-3 levels were unchanged. **K** Total ubiquitinated protein levels were lower after DDp53 overexpression compared to the contralateral control muscle. **L** Free ubiquitin levels were not different between the DDp53 and the control tibialis anterior muscle. Individual data points represent individual animals. Squares represent the leg electroporated with DDp53, circles the contralateral control leg of the same animals. Western blot images show three representative bands from each condition. Both representative pictures stem from the same membrane and exposure time. All western blots were normalized to total protein per lane. Original, unaltered images of all membranes and the gels that they were normalized to can be found in the data supplement. *n* = 12, * indicates a *p*-value of <0.05.

Since measures of muscle protein synthesis were increasing, whereas the muscle fiber CSA was decreasing, we next examined signaling pertaining to proteolysis, inflammatory pathways, and cell death in muscle. Overexpression of DDp53 did not change phospho-ULK1 (Ser757) levels (*p* = 0.52), which is important in the regulation of autophagy and primarily phosphorylated and inhibited by mTORC1 (Fig. 6A) [52]. Total FOXO3a, a central hub for the control of the autophagy, lysosomal, and ubiquitin-proteasomal degradation pathways in muscle [53, 54], increased 39% (*p* < 0.05) in the DDp53 treated compared to the control TA (Fig. 6B). The autophagy related protein Atg7 did not change (*p* = 0.32) with DDp53 compared to the control condition (Fig. 6C). DDp53 increased p62, a substrate that links autophagy to the ubiquitin-proteasome system [55, 56], by 55% (*p* = 0.06) but this did not reach statistical significance (Fig. 6D). Neither LC3I, nor LC3II or LC3II to I levels changed significantly (*p* = 0.39, *p* = 0.96 and *p* = 0.4, respectively) with the DDp53 treatment compared to the control condition (Fig. 6E–G). To investigate oxidative and inflammatory signaling, we looked at chaperones and proteins central to the NF-κB pathway. HSP27 protein levels did not differ (*p* = 0.78) between the DDp53 treated and the contralateral control (Fig. 6H). Phosphorylated NF-κB p65 (Ser 536) levels, a marker of inflammation, were 24% higher (*p* = 0.16) in the DDp53 TA compared to the contralateral control TA without reaching statistical significance (Fig. 6I). Similarly, mean caspase-3 levels, a hallmark protein for apoptosis, were 23% higher (*p* = 0.27) in the DDp53 TA compared to the contralateral control TA without reaching statistical significance (Fig. 6J). Interestingly, despite increased FOXO3a in the DDp53 TA, total ubiquitinated protein levels were 19% lower (*p* < 0.05) than in the contralateral control TA (Fig. 6K). Free ubiquitin levels did not differ (*p* = 0.9) between DDp53 and the contralateral control (Fig. 6L).

### The effect of DDp53 on proteasomal activity in skeletal muscle

**Fig. 7:**
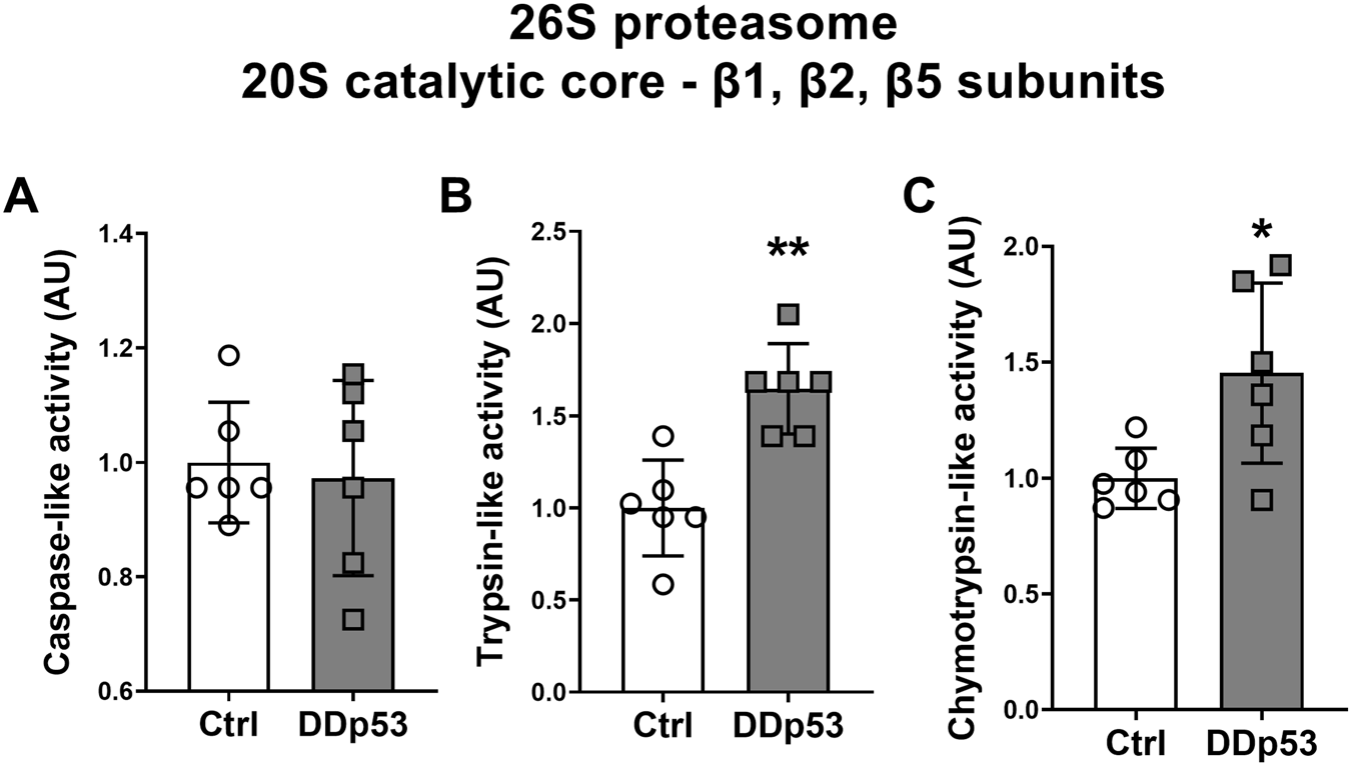
Activity of the individual subunits of the 20S catalytic core complex of the 26S proteasome with chronic DDp53 overexpression. **A** Activity of the caspase-like β1 subunit was unchanged between both legs. **B** Trypsin-like activity of the β2 subunit was increased in the DDp53 compared to the control leg. **C** Chymotrypsin-like activity of the β5 subunit was increased with DDp53 compared to the control side. Individual data points represent the results from each animal. Squares represent the leg that was electroporated with DDp53, circles the contralateral control leg of the same animals. *n* = 6, * indicates a *p*-value of <0.05, ** indicates a *p*-value of <0.01.

Since the ubiquitin-proteasome system is responsible for the majority of protein breakdown in eukaryotic cells [57] and to gain a more precise understanding of the effect of DDp53 on protein degradation and the change in muscle fiber CSA, we performed a direct measure of proteasomal-activity of the 26S proteasome and the catalytic subunits of its 20S core complex [33]. The caspase-like activity of the 26S proteasome β1 subunit did not change (*p* = 0.56) in the DDp53 treated leg (Fig. 7A). This was in keeping with the lack of a change in caspase-3 protein levels described above (Fig. 6J). Overexpression of DDp53 in skeletal muscle did, however, cause a 65% increase (*p* < 0.01) in trypsin-like activity of the 26S proteasome β2 subunit in the DDp53 leg compared to the contralateral control (Fig. 7B). Additionally, chymotrypsin-like activity of the 26S proteasome β5 subunit increased by 45% (*p* < 0.05) in the electroporated DDp53 tibialis anterior compared to the contralateral control muscle (Fig. 7C). These data suggest that while levels of total ubiquitination were similar between both conditions (Fig. 6L), the activity of the ubiquitin-proteasome system and specifically the trypsin-like β2 and chymotrypsin-like β5 subunits were increased with DDp53 overexpression and are likely to have driven the decrease in ubiquitinated proteins, muscle mass and fiber CSA present in the DDp53 electroporated leg.

### Association between DDp53 expression, muscle phenotype and protein levels

**Fig. 8:**
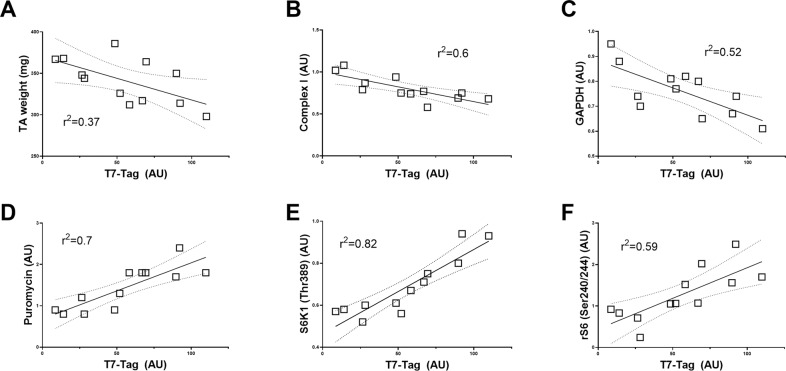
Linear regression analysis between T7-tag protein levels, skeletal muscle phenotype, and the regulation of muscle protein synthesis- and degradation in DDp53 overexpressing tibialis anterior muscle. **A** Muscle mass, **B** Mitochondrial complex I protein, and **C** GAPDH in the DDp53 electroporated leg correlate inversely with DDp53 protein levels. In contrast, **D** muscle protein synthesis via puromycin, **E** mTORC1 activity (phospho-S6K1 (Thr389) levels), and **F** S6K1 activity (phospho-rS6 (Ser240/244) levels) are positively associated with DDp53.

To better understand the relationship between DDp53 and the physiological changes we observed from Figs. 1–7, we performed a linear regression analysis between T7-tag levels (tagging the DDp53 protein) and phenotypical features like muscle mass and muscle cross-sectional area as well as various biochemical measures in the TA that was electroporated with DDp53. This was to specifically investigate the changes within the muscle that contained the DDp53 plasmid rather than to compare the differences between the control muscle (mCherry) and the DDp53 overexpressing muscle. Muscle mass of the TA in the DDp53 electroporated leg correlated (r^2^ = 0.37; *p* < 0.05) in a negative manner with T7-tag protein levels (Fig. 8A), supporting the notion that there is a relationship between the amount of DDp53 present and the extent of muscle wasting occurring. Further, T7-tag protein levels in the electroporated DDp53 TA were closely, negatively associated (r^2^ = 0.6; *p* < 0.01) with complex I protein levels in the TA (Fig. 8B). This suggests that the inhibition of wildtype p53 through overexpression of DDp53 is associated with a decrease in mitochondrial function as has been reported previously for whole-body p53 knockout models [11–14]. Similarly, GAPDH protein levels were negatively associated (r^2^ = 0.52; *p* < 0.01) with T7-tag levels (Fig. 8C). Protein synthesis rates via puromycin protein levels were closely, positively associated (r^2^ = 0.7; *p* < 0.001) with T7-tag protein levels in the DDp53 electroporated leg (Fig. 8D). Interestingly, despite the negative effect of DDp53 on phospho-S6K1 (Thr389) (Fig. 5B), protein levels of phospho-S6K1 (Thr389) were closely correlated (r^2^ = 0.82; *p* < 0.0001) with T7-tag levels (Fig. 8E). In line with that, downstream of S6K1, S6 (Ser240/244) phosphorylation correlated positively (r^2^ = 0.59; *p* < 0.01; Fig. 8F) with T7-tag levels as well. These relationships indicate that while overall phospho-S6K1 protein levels decreased with DDp53 overexpression, greater DDp53 protein content is associated with translational initiation and increased protein synthesis. This would be consistent with other recent reports that found even small increases in mTORC1 activity can suffice to maintain muscle homeostasis for multiple weeks [58, 59].

## Discussion

We investigated the effect of overexpression of dominant-negative p53 in skeletal muscle. We hypothesized that p53 would act as a molecular brake to muscle fiber size and that inhibiting wild-type p53 function through transfection of a dominant-negative mutant p53 into skeletal muscle would facilitate myofiber growth. Contrary to our hypothesis, we found that overexpression of DDp53 resulted in a decrease in muscle mass and fiber size that was associated with significant changes to the cell structure, oxidative metabolism, mTORC1 signaling, and protein turnover.

p53 is a thoroughly investigated growth/tumor suppressor. Increased copies of the TP53 gene are associated with increased lifespan and a decreased susceptibility to cancer across different species [8–10]. Mutations in p53 are found in numerous cancers across a variety of species including mice, rats, and humans [16, 18]. Since p53 is important in limiting cell growth, we hypothesized that inhibiting p53 locally in skeletal muscle could improve muscle fiber size. By analogy, the genetic or pharmaceutical inhibition of myostatin and the TGFβ pathway result in up to a 4-fold increase in muscle mass [5, 6].

Contrary to our hypothesis, the inhibition of wild-type p53 using a mutant p53 that exerts a dominant-negative effect (i.e., DDp53) decreased muscle mass by 4% and myofiber cross-sectional area by 43% in the DDp53 electroporated leg compared to the contralateral control leg. Histological analysis further revealed excessive fiber damage, indicators of cell death, and remodeling. In line with the histological analysis, we found various proteins associated with cytoskeletal integrity to be affected by DDp53. While we have not confirmed the localization of these proteins histologically, the findings are consistent with our previous observations in aged and diseased muscle, where we found the loss of dystrophin and increased dysferlin and annexin A2 to be markers of increased membrane damage in rat muscle [38], which could be attenuated through restoration of dystrophin protein levels [60].

Beyond gross histological changes, we observed a trend for decreased dystrophin levels, and increased dysferlin and annexin A2 levels with DDp53, which are consistent with decreased membrane integrity, and increased damage and repair of the sarcolemma. In addition, we found an increase in the content of the slow myosin heavy chain isoform in muscle overexpressing DDp53. Rather than a transformation of fast to slow myofibers, this likely suggests a fiber-type specific loss of the fast type 2 fibers as is the case in many muscle wasting scenarios [39]. We also observed an increase in pro-Col1α1 in DDp53 treated muscle. The increase in pro-Col1α1 protein could explain some of the fibrotic changes we observed on the histological level. However, whether this is a direct effect of p53 inhibition or an indirect effect of muscle deterioration has yet to be determined. Surprisingly, we did not see a change in protein levels of mLIM which has been reported to be a sensor of mechanical stress that associates with the cytoskeleton and the z-disc [41, 42]. However, we did find a correlation between mLIM and T7-Tag levels in the DDp53 electroporated muscle, indicating that mLIM is still positively regulated by the presence of DDp53 or its negative effect on muscle homeostasis (Supplemental Fig. 1).

One key function of p53 is its effect on the maintenance of mitochondrial function and oxidative metabolism. Previous investigations in whole-body knockout mice have found deleterious effects of the absence of p53 on mitochondrial biogenesis, DNA content, enzyme activity, and endurance performance [11–13]. However, muscle-specific knockout of p53 neither alters mitochondrial content and enzyme activity, nor affects muscle loss in the context of aging [14, 15]. We found a robust decrease in mitochondrial complex I (NDUFB8), mitochondrial complex V (ATP5A), and GAPDH levels in DDp53 electroporated muscle three weeks after transfection. We also found that the decrease in NDUFB8 and GAPDH closely correlated with DDp53 protein levels in the electroporated leg. This suggests that impairing the function of wild-type p53 through the overexpression of a dominant-negative mutant p53 causes a decline in mitochondrial function and oxidative metabolism that more closely resembles the findings from whole body- than muscle-specific p53 knockouts.

The growth-suppressing effects of p53 via the induction of apoptosis are well documented. However, we found that inhibiting p53 using DDp53 also results in decreased muscle cell size. Traditionally a decrease in myofiber size is thought to be governed by the balance between myofibrillar protein synthesis and breakdown, where either a decrease in synthesis, an increase in breakdown, or both occurring simultaneously result in a reduction in size. In contrast to this model, we found that overexpressing DDp53 in skeletal muscle induced substantial fiber atrophy that was associated with a 40-65% increase in proteasomal activity and a concomitant 43% increase in muscle protein synthesis. Interestingly, we found decreased mTORC1 activity (phospho-S6K1 Thr389 levels) in the DDp53 leg. Despite this decrease in phospho-S6K1 protein levels, S6K1 phosphorylation did correlate with DDp53 levels and protein synthesis (Fig. 8). Therefore, it is possible that there was still sufficient mTORC1 activity to increase protein synthesis and that small increases in mTORC1 activity supported the increase in protein synthesis in the DDp53 treated muscle. The hypothesis that low mTORC1 activity may suffice to sustain protein synthesis is in line with recent work from the Blaauw laboratory showing that low levels of mTORC1 signaling, after the deletion of Raptor, are able to maintain muscle homeostasis for weeks [58, 59]. Further, our previous work showed that 30 min after endurance exercise there is a 50% decrease in mTORC1 activity even though myofibrillar protein synthesis is elevated in a rapamycin-sensitive manner [61]. Together, these data suggest that mTORC1 may still be important for the increase in protein synthesis in the DDp53 electroporated leg.

An additional explanation for the increase in protein synthesis despite an overall decrease in mTORC1 activity could be the activation of mTORC1-independent pathways. Recent observations by our laboratory and others [28, 62] showed that even with rapamycin treatment, protein synthesis following resistance exercise is able to reach similar increases compared to untreated muscle, likely through an mTORC2-dependent pathway [63].

Finally, it is also possible that three weeks into DDp53 overexpression, acute levels of specific proteins do not reflect chronic changes to the muscle phenotype but rather a compensatory attempt to maintain homeostasis. Consistent with this, we measured a 65% increase in 26S proteasome β2 and 45% increase in β5 activity in the DDp53 leg, but a decrease in polyubiquitinated proteins and increased levels of the autophagy substrate p62. The decrease in polyubiquitinated proteins is likely to be a function of increased clearance rates since it is known that blocking the ubiquitin proteasome system through bortezomib leads to an increase in polyubiquitinated proteins [64–66]. While p62 protein levels are notoriously difficult to interpret, an accumulation could hint at a decrease in activity of the ubiquitin proteasome system [55, 56] and/or autophagic flux [67, 68]. Given our observation that proteasome activity was increased, the latter appears more likely and is supported by growing evidence that autophagy is impaired in many scenarios of muscle loss whereas restoring the autophagy-lysosome system helps maintain muscle mass and integrity [69–73]. Although the importance of p53 in regulating apoptosis is well described, its role in autophagy is more ambiguous [74–76]. Despite this, it is possible that inhibiting wild-type p53 had a negative effect on autophagy that contributed to muscle loss in our study. Overall, recent reports indicate that putative dissociations between anabolic signaling, protein synthesis and muscle mass may be a common phenomenon in various types of muscle wasting [69, 70, 77–79], illustrating the difficulty in relating transient processes like molecular signaling to muscle protein turnover and changes in the phenotype.

Accordingly, a limitation of our study is that we did not follow the molecular signaling over multiple time points, making the distinction between acute and chronic signaling challenging. However, we conducted a pilot experiment where we collected TA muscles 7 days after electroporation. In these muscles we did not find any significant changes in the phenotype despite a robust increase in DDp53 (Supplemental Figs. 2 and 3). Therefore, we think that the changes observed at 21 days reflect chronic adaptations of skeletal muscle to the overexpression of DDp53. Another limitation is that we only investigated global muscle protein synthesis and proteasomal activity. Therefore, direct insight into which proteins are being synthesized and degraded is not possible. Despite this, some mechanistic inferences are possible. For example, assessing global protein synthesis in muscle via the SUnSET, utilizing the tyrosine aminoacyl-transfer RNA analog puromycin [29], has shown great correspondence with the synthesis of skeletal muscle- and contractile proteins in vitro and in vivo compared to other standard methods such as S^35^ incorporation [30, 80]. This is likely owed to the fact that contractile proteins comprise most of the total protein pool in skeletal muscle tissue [81, 82]. Therefore, our findings of increased global muscle protein synthesis in the DDp53 overexpressing leg are likely to reflect increased contractile protein synthesis. Similarly, it could be inferred that the increase we found in proteasomal activity in the DDp53 electroporated leg indicates degradation of the contractile apparatus. This is supported by the observation that total muscle mass, and more importantly, individual fiber cross-sectional area and diameter were decreased in the DDp53 overexpressing muscle. However, the possibility of proteins outside of the contractile machinery being primarily synthesized and degraded cannot be conclusively excluded with our current approach. If the non-contractile fraction were disproportionately affected by synthesis and degradation, this could alter the total protein pool and bias the comparison of individual protein levels between the conditions. Finally, it is possible that the overexpression of DDp53 in the tibialis anterior affected cell types other than skeletal muscle, such as neuronal cells resulting in damaged motor end plates. This indirect effect could explain why the phenotype in our experiments is more severe and more closely resembles findings from the whole body rather- than muscle-specific p53 knockout models.

Future studies that explore how DDp53 impairs wild-type p53 function and why this leads to poor proteostatic control and tissue loss in muscle need to be carefully designed. For example, a time-course analysis utilizing techniques such as stable isotope tracing and targeted proteomics could inform us about how and when p53 manipulation impacts the turnover of proteins, and which proteins specifically are affected by it. This would allow to delineate how wild-type p53 governs muscle fiber size as well as test the hypothesis that the increase in protein synthesis we observed indeed reflects the turnover of contractile proteins rather than those needed to initiate and execute the remodeling process. However, based on the results of our study we need to conclude that decreasing wild type p53 activity through DDp53 does not currently appear to be a promising therapeutic strategy to improve muscle mass and function.

In summary, we found that inhibition of wild-type p53 in skeletal muscle through electroporation of a dominant-negative mutant p53 results in decreased muscle mass, fiber cross-sectional area, and -fiber diameter as well as increased remodeling more typical of myopathic muscle. These phenotypical changes were accompanied by a distinct molecular signature. This signature is characterized by increased concentrations of proteins associated with membrane damage and fibrosis, decreased levels of oxidative phosphorylation, and increased proteasomal activity that is accompanied by elevated protein synthesis. In conclusion, targeted impairment of wild-type p53 function in skeletal muscle results in deterioration of muscle integrity on a structural, metabolic, and proteostatic level.

## Supplementary information

Supplemental Material

Reproducibility Checklist

## Data Availability

All raw experimental data supporting the conclusions of this article will be made available upon request to the corresponding author.
