# Supplementary material for: Dominant-negative p53-overexpression in skeletal muscle induces cell death and fiber atrophy in rats
Source: Cell Death Dis. 2022 Aug 17;13(8):716. doi: 10.1038/s41419-022-05160-6 (PMC9385859; doi:10.1038/s41419-022-05160-6)
Supplement: Supplementary file 1 — Supplemental Material [file 41419_2022_5160_MOESM1_ESM.docx]

**Supplemental Figure 1.**


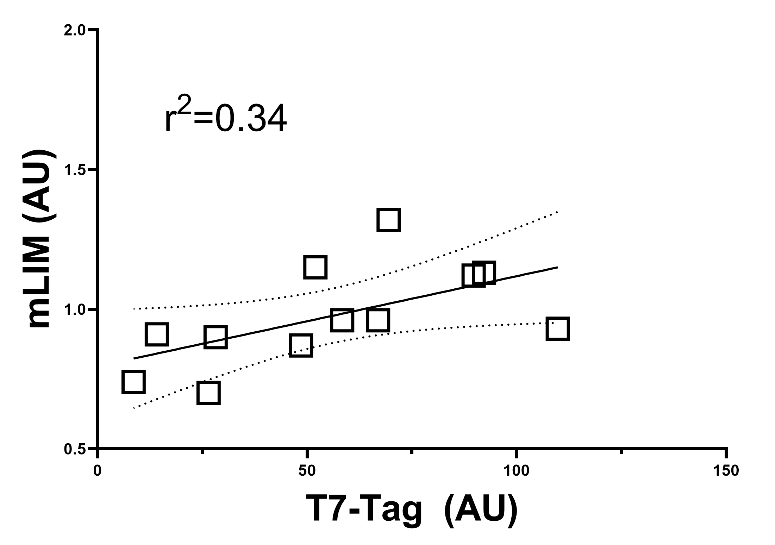


Supplemental Figure 1 description: Protein levels of muscle-specific LIM (mLIM/CSRP3) are significantly correlated with T7-tagged DDp53 protein levels (p<0.05).

**Supplemental Figure 2.**


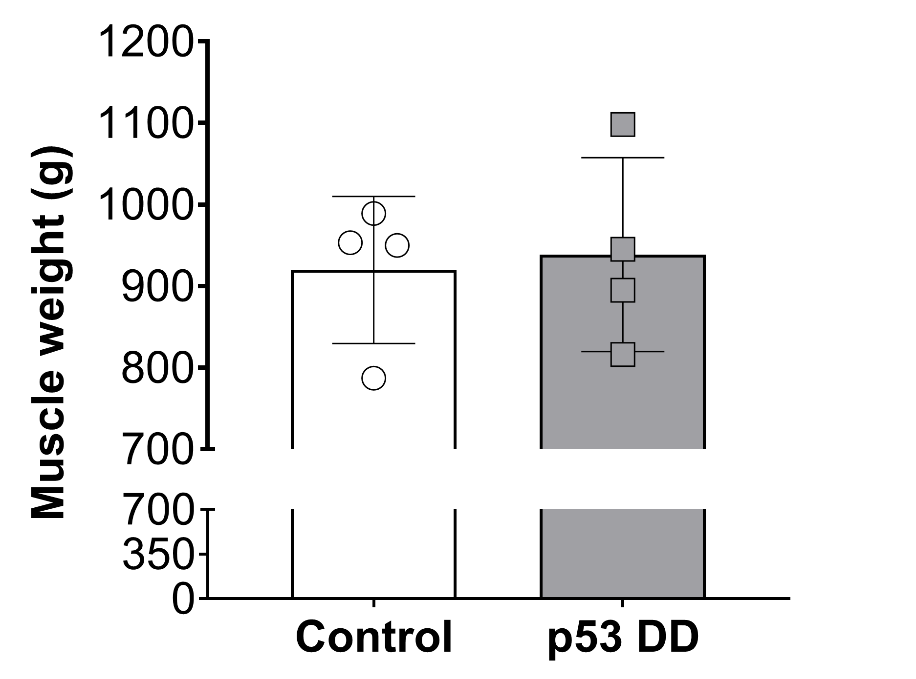


Supplemental Figure 2 description: Muscle mass of the tibialis anterior of rats after 7d of electroporation (“p53 DD”) and in the contralateral control leg of the same animals (“Control”). Squares and circles represent individual data points from the same animals in the electroporated and contralateral control leg, respectively. There was no change or trend for a change in muscle mass at this timepoint. *n*=5.

**Supplemental Figure 3.**


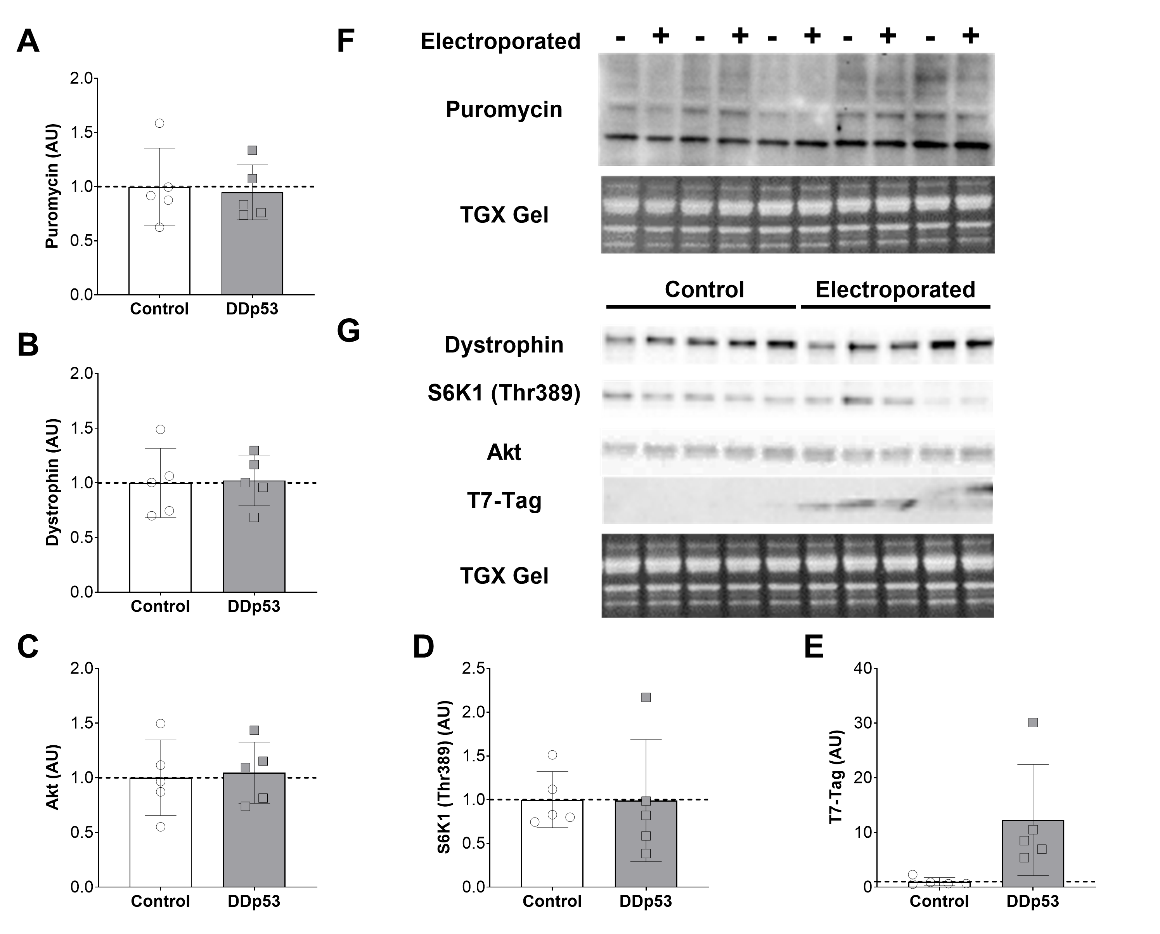


Supplemental Figure 3 description: Protein levels of puromycin (A), dystrophin (B), total Akt (C), phospho-S6K1 (Thr389) and the T7-epitope tag (indicative of DDp53 protein levels) (E) in rat tibialis anterior after 7d of electroporation (“DDp53”, gray bar, and squares) and in the contralateral control leg (“Control”, white bar, and circles) of the same animals. No change in protein synthesis via puromycin incorporation or any of the other proteins was evident, despite DDp53 protein levels (via T7-tag) being 11.3-fold as high in the electroporated compared to the control leg. (F) shows a representative image of the puromycin western blot quantified in (A), with lanes alternatingly being loaded with muscle lysate from the control or the electroporated tibialis anterior of the rats. Similar to the representative picture of the puromycin western blot, (G) shows representative images of the quantification of the proteins from (B) to (E). The first five lanes were loaded with control muscle lysates (“Control”) before the next five lanes were loaded with DDp53 electroporated samples (“Electroporated”) being loaded. *n*=5.
